# Evaluating the Intoxicating Degree of Liquor Products with Combinations of Fusel Alcohols, Acids, and Esters

**DOI:** 10.3390/molecules23061239

**Published:** 2018-05-23

**Authors:** Jia Xie, Xiao-Fei Tian, Song-Gui He, Yun-Lu Wei, Bin Peng, Zhen-Qiang Wu

**Affiliations:** 1 School of Biology and Biological Engineering, South China University of Technology, Guangzhou 510006, Guangdong, China; ncuskxiejia@163.com (J.X.); xtien@scut.edu.cn (X.-F.T.); pengbin163163@163.com (B.P.); 2 Guangdong Jiujiang Distillery Co., Ltd., Foshan 528203, Guangdong, China; nh1314@126.com (S.-G.H); wylqz@yahoo.com.cn (Y.-L.W.)

**Keywords:** alcohol consumption, liquor products, fusel alcohols, intoxicating degree, mice model

## Abstract

To investigate the effects of fusel alcohols on the intoxicating degree of liquor products, formulated liquors (FLs) were prepared by blending 1-propanol, isobutanol, and isoamyl alcohol with ethanol, organic acids, and corresponding ethyl esters to simulate the formula of traditional Chinese liquors. The prepared FLs were submitted for evaluation of their intoxicating degree (ID). The results showed that the fusel alcohols had a biphasic effect on the IDs of the FLs, depending on the comprehensive coordination of the characteristic minor components. The importance of the suitable ratio of alcohols/acids/esters (RAAE) on the IDs was also revealed. Under an optimal ratio level, the fusel alcohols exhibited negligible effects on the IDs of the FLs. Moreover, the ratio of isoamyl alcohol to isobutanol (IA/IB) showed a strong positive correlation to the IDs of the FLs. This study lays a foundation for the potential application in producing low-ID liquor.

## 1. Introduction

Alcoholic beverages are widely consumed throughout the world. The beneficial and adverse effects of alcohol consumption have drawn widespread attention. Previous studies have shown that light to moderate alcohol consumption can exert a series of protective effects [1]. Compared to nondrinkers, people who drink regularly and moderately tend to have better insulin sensitivity and reduced overall mortality [2], as well as lower risks for cardiovascular disease [3], diabetes [4,5], dementia [6], and obesity [7]. However, excessive consumption of alcohol is often associated with severe organ damage and a range of hangover symptoms, such as dizziness, headache, nausea, fatigue, and muscle pain [8]. In addition, the overconsumption of alcohol can lead to a burden of disease and dysfunction, such as alcoholic hepatitis [9], cardiomyopathy [10], hypertension [11], and stroke [12], as well as wide-ranging neurological damage, including Wernicke encephalopathy, dementia, delirium tremens, and peripheral polyneuropathy [13]. The primary common risk factors of alcohol abuse have been considered to be oxidative stress and acetaldehyde [14]. In the human body, the hepatic oxidation pathway detoxifies most alcohol by oxidizing it to acetaldehyde and further to acetate via the enzymes alcohol dehydrogenase (ADH) and acetaldehyde dehydrogenase (ALDH), respectively [15].

In the process of liquor fermentation, many microbial metabolites are co-produced with ethanol, and thus, a typical commercial alcoholic liquor product contains 98% (*v*/*v*) ethanol and water and 2% minor components, including fusel alcohols, esters, and organic acids [16]. For example, the fusel alcohols produced from amino acids by yeast are present in most alcoholic beverages [17]; these generally include 1-propanol, 1-butanol, 2-butanol, isobutanol, and isoamyl alcohol. As they are generally regarded as “flavoring agents”, European legislation even demands a minimum content of fusel alcohols in certain spirit products [18]. In addition, Chinese legislation first raised the maximum limit for total fusel alcohols (calculated from both isobutanol and isoamyl alcohol) from 1.5 to 2.0 g/L but subsequently canceled the limit for fusel alcohol content through revising the hygienic standard for distilled liquor and formulated liquor [19]. Although fusel alcohols are considered to be safe flavoring components, their contribution to the intoxicating effects and quality of liquor products warrants investigation. In fact, fusel alcohols could be a key contributor to the intoxicating effects, as a result of their acute toxicity and neurotoxic effects [18]. Studies have revealed the high possibility of fusel alcohols contributing to the intoxicating effect in certain liquor products [20]. Furthermore, other liquor components, including organic acids and esters, may mediate protective effects on human alcohol metabolism and may therefore attenuate the intoxicating effects.

To quantify the intoxicating effect of liquor products, Wu et al. developed a method to evaluate the intoxicating degree (ID) using a mouse model [21]. The ID is a reliable index that closely reflects the quality of liquor products by integrating the contribution of multiple chemical components to the overall intoxicating effect. It is convenient and practical to investigate the role of fusel alcohols on the intoxicating effects of liquor products on the basis of the ID evaluation method.

In this study, the intoxicating effects of formulated liquors (FLs) containing various fusel alcohols were investigated to address the topic of fusel alcohol in liquor quality control. The fusel alcohols 1-propanol, isobutanol, and isoamyl alcohol, which are the primary fusel alcohols in Chinese high-alcohol-volume liquors, were selected and blended with ethanol, organic acids, and their ethyl esters to prepare the FLs. The IDs of the resulting FLs were investigated and analyzed in detail.

## 2. Results and Discussion

### 2.1. Determination of Characteristic Compounds of Commercial Chinese Liquors for the Preparation of FLs

The quantity and proportion of the characteristic components in liquor play a vital role in the aroma and quality of liquor [22,23]. Fusel alcohols were found to have concentrations of pure ethanol ranging between 0.8 and 2.5 g/L in most Chinese liquors [24]. Comparably, fusel alcohols were found to have concentrations of pure ethanol of approximately 4.0 g/L in most imported liquors [18]. Additionally, the isoamyl alcohol to isobutanol (IA/IB) ratio was also found to differ among liquor products. This difference may be due to a lack of a standard procedure for liquor production and the variable raw materials used in different regions [25]. The characteristic compounds, including alcohols, acids, esters, acetals, ketones, aldehydes, and heterocyclic compounds, have been identified in commercial Chinese liquors [16,26]. In this study, 13 commercial Chinese liquors representing 6 flavor types, Jiang-flavor, strong aromatic, mild aromatic, rice aromatic, Te-flavor, and Feng-flavor, were selected for analysis of the concentration and proportion of these characteristic compounds (Table 1). The compounds with contents of ≥10 mg/100 mL included four organic acids (acetic acid, l-lactic acid, *n*-butyric acid, and *n*-hexanoic acid), four ethyl esters (ethyl acetate, ethyl lactate, ethyl butyrate, and ethyl hexanoate), three fusel alcohols (1-propanol, isobutanol, and isoamyl alcohol), methanol, acetal, and acetaldehyde. In general, liquor products with the same flavor type shared a similar ratio of alcohols/acids/esters (RAAE). The total fusel alcohol content was approximately 0.5 g/L in most of the liquors, which had varying IA/IB ratios. Methanol, acetal, and acetaldehyde were also common components in the liquors, but they were not selected for inclusion in the FLs for this study because of their prominent toxicity and the toxicity of their metabolites [18,27].

### 2.2. Effect of RAAE on the IDs of FLs

Because compositions differ among liquor products, it was indispensable to assess the effect of the RAAE on the IDs of the FLs prior to a further study on fusel alcohols. On the basis of the FL design (Table 2), five FLs were prepared for the ID evaluation. The results showed that the optimal RAAE and the pessimal RAAE were 1:2:3 and 1:1:1.5, which led to the lowest ID and the highest ID, respectively (Table 3). Compared to A2, the ID of A3 showed a marked decrease from 1.270 ± 0.031 to 1.102 ± 0.012 (*p* < 0.01) when the ester content increased from 5.0 to 7.0 g/L. Similarly, the ID of A5 significantly decreased from 1.270 ± 0.031 to 1.120 ± 0.089 (*p* < 0.01) when the acid content increased from 1.5 to 3.0 g/L. Moreover, A3, A4, and A5 resulted in remarkably low IDs compared to A1 (*p* < 0.01) when the content of acids and esters increased simultaneously. This finding indicated that the increase in both acids and esters could reduce the IDs of FLs. Similarly, previous studies have reported that moderate concentrations of acetic acid and acetate may mediate protective health effects by activating the hepatic AMPK, which induces the synthesis of certain long-lived proteins for cardiovascular protection [28,29], hyperglycemic effect [30], and fat oxidation [31,32]. Acetic acid and corresponding ethyl acetate are major components in FLs. Therefore, the increase in acids and esters in FLs could play a vital role in mitigating the drunkenness reaction.

However, a contrasting result showed that the IDs of the FLs could increase when the acid and ester content increased, by a comparison of A5 and A4. Liquor quality was considered to be determined by the characteristic components and their overall coordination. Therefore, the metabolic efficiency for liquor products containing an equivalent content of ethanol could be different in the human body. Other studies have suggested that top-brand liquor products such as Moutai from China would not harm the human liver, owing to a balanced composition of the minor compounds [33,34]. It could be concluded that liquor quality also depends on an integrated effect of the characteristic components. Thus, the proper RAAE may play a crucial role in the intoxicating effects of the liquor products.

### 2.3. Effect of Fusel Alcohol Concentration on the IDs of FLs

**Figure 1:**
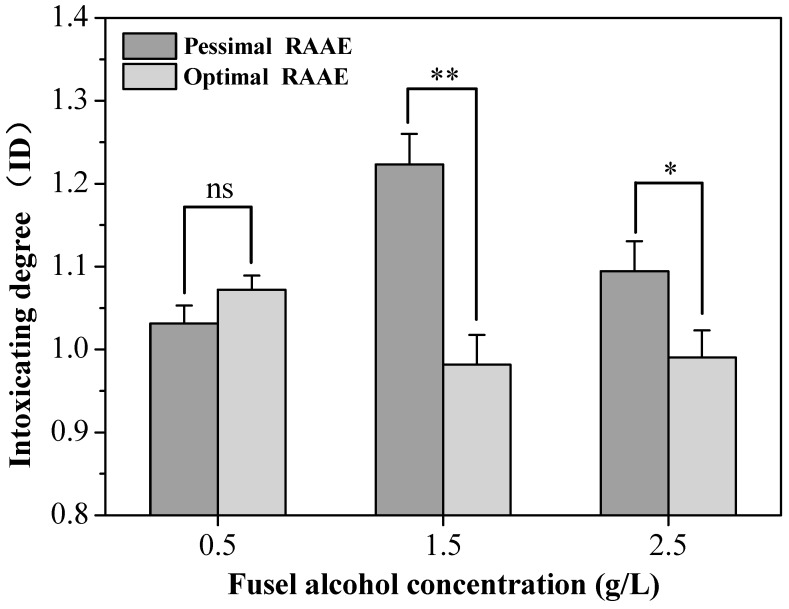
The effects of fusel alcohol concentration on the intoxicating degree (ID) of formulated liquors (FLs). Each value is expressed as the mean ± standard deviation (*n* = 3). Levels of statistical significance are compared among FLs with same fusel alcohol concentrations (ns: not significant; * *p* < 0.05; ** *p* < 0.01; RAAE: the ratio of alcohols/acids/esters).

Fusel alcohols are essential flavoring compounds, but a high content of fusel alcohols in alcoholic beverages has been shown to possibly be responsible for higher toxicities and hangover symptoms in humans [20]. To evaluate the effects of fusel alcohol content on the IDs of FLs, three concentrations of fusel alcohols were prepared with both the optimal RAAE and the pessimal RAAE (Table 2, experiment B). When the fusel alcohol content was approximately 16.7% with the optimal RAAE, the IDs of FLs B1, B2, and B3 were lower, ranging from 0.9816 to 1.0885 (Figure 1).

In contrast, the FLs B4, B5, and B6 had higher IDs ranging from 1.0315 to 1.2234 when the fusel alcohols accounted for approximately 28.6% with the pessimal RAAE. The average IDs of the FLs with the optimal RAAE were lower than those with the pessimal RAAE. This result supported the previous findings that fusel alcohols were closely related to the intoxicating effects of alcoholic beverages [35,36,37]. On the other hand, this finding also indicated that a proper RAAE could diminish acute alcohol intoxication (AAI) or reduce the volatility of the IDs in FLs.

In addition, a distinctly inverted “U” curve was observed, indicating that 1.5 g/L was the most effective level at which fusel alcohols contributed to the increased ID under the pessimal RAAE. In contrast, a negligible “U” curve showed that 1.5 g/L was the most effective level at which fusel alcohols decreased the ID under the optimal RAAE (Figure 1). It has been considered that the potential toxicities of the liquors could be promoted by the interaction between ethanol and fusel alcohols [38]. It has also been reported that a high fusel alcohol content could inhibit the tricarboxylic acid cycle. As the result, the organic acids derived from the alcohols would not be oxidized but would accumulate and subsequently depress the animal [39]. In that case, 2.5 g/L of fusel alcohols should be the most effective level to cause differences in ID. However, the role of fusel alcohols in the intoxicating effects of liquor is still disputed. It has been demonstrated that the class III ADH (ADH3) plays a key metabolic role during the AAI by compensating for the reduced contribution of class I ADH (ADH1) [40,41], the microsomal ethanol oxidizing system (MEOS) [42], and catalase [43]. The effect of ADH3 on the ethanol metabolism system could be activated by hydrophobic substances, such as alcohols (trifluoroethanol and tert-butanol), fatty acids, amino acids (trichloroacetic acid, stearic acid, phenylalanine, and norleucine), and fatty acid amides (butyramide, valeramide, capronamide, and oleamide) [44,45]. As a ubiquitous ADH of ancient origin [46], ADH3 is the only ADH that has been detected in brain tissue [47]. Therefore, ADH3 could be protective against AAI through reducing the detrimental effects of ethanol on the brain. On the basis of these facts, the hydrophobic fusel alcohols from the FLs could promote the activation of ADH3 if the hydrophobicity were increased. The elimination of the ethanol was accelerated, reducing the drunkenness reaction. Moreover, the suitable contents of the acids and esters in the FLs could also exhibit sufficient protective effects to attenuate the aggravation of IDs caused by a high content of fusel alcohols.

While the effect of fusel alcohols on liquor IDs remains controversial, this study showed that a proper RAAE could effectively adjust the IDs, despite the fact that a high fusel alcohol content could possibly raise the toxicity of the liquor products.

### 2.4. Effect of the Ratio of Isoamyl Alcohol to Isobutanol (IA/IB) on the IDs of FLs

**Figure 2:**
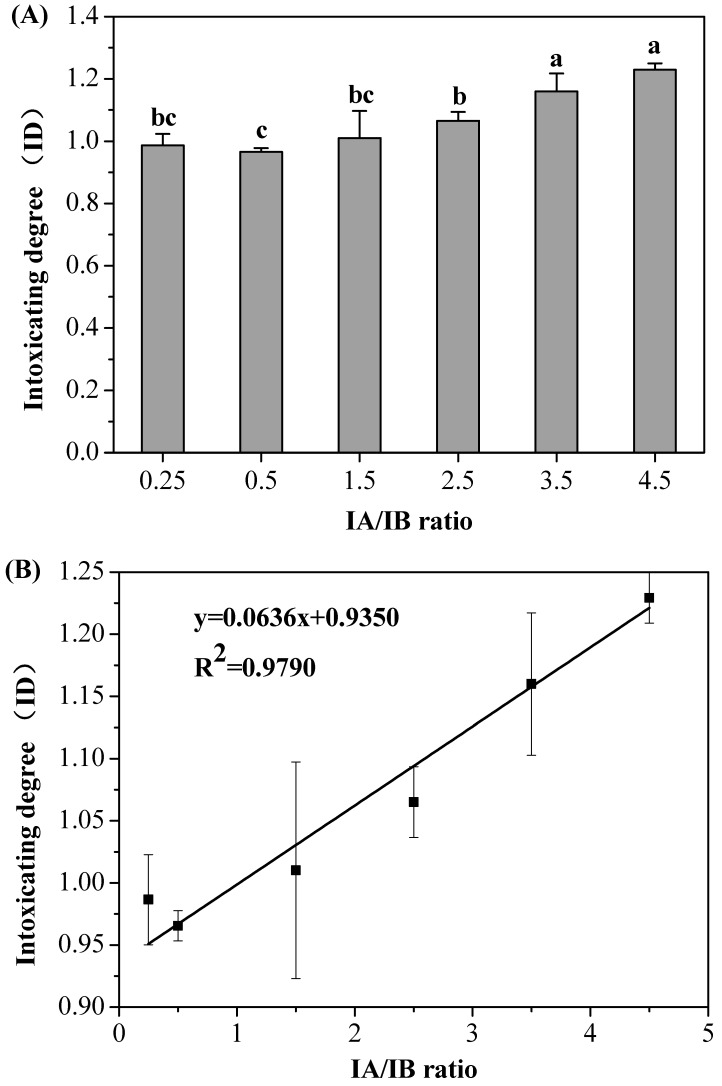
(**A**) The effects of isoamyl alcohol to isobutanol (IA/IB) ratio on the intoxicating degree (ID) of formulated liquors (FLs); and (**B**) the relationship between the IA/IB ratio and ID. Each value is expressed as the mean ± standard deviation (*n* = 3). Different letters (a, b, c) indicate significant differences in the IDs of FLs. IA: isoamyl alcohol; IB: isobutanol; IA/IB: isoamyl alcohol/isobutanol.

Isoamyl alcohol has always co-existed with isobutanol in alcoholic beverages [17]. These two alcohols dominated the fusel alcohols in the liquor products (Table 1). Owing to different fermentation substrates and different fermenting methods, the IA/IB ratio showed significant differences [25,48]. In addition to affecting the liquor aroma and flavor, the proper IA/IB ratio was also thought to contribute to the health-protective function of Chinese liquors. In this study, the results showed that the IDs of the FLs increased gradually with the increase in the IA/IB ratio from 0.25 to 4.5 (Figure 2A), and a strong correlation (*R*^2^ = 0.9790) between the ID and the IA/IB ratio was observed (Figure 2B). This finding indicated that the drunkenness reaction from the FLs could be aggravated with a high IA/IB ratio.

It was previously reported that fusel alcohols could have a direct and long-lasting effect on the central nervous system [49]. An increase in the toxicity effect of the fusel alcohols was found to be related to the length of the carbon chain [50,51]. Here, an increase in the IA/IB ratio resulted in the aggravation of intoxicating effects, in agreement with previous reports. The oxidization rates of isoamyl alcohol and isovaleraldehyde were lower than those for isobutanol and isobutyraldehyde. Additionally, isovaleraldehyde was the most potent inhibitor of the oxidization of various mitochondrial substrates, including acetaldehyde [52]. The more severe intoxication resulting from isoamyl alcohol could be explained by the inhibition of the metabolism of ethanol and acetaldehyde. Accordingly, it is concluded that the IA/IB ratio should be restricted for a low ID.

## 3. Materials and Methods

### 3.1. Chemical Reagents

Ethanol (>99.8%), 1-propanol (≥99.8%), isobutanol (≥99.5%), isoamyl alcohol (≥99.8%), acetic acid (≥99.8%), l-lactic acid (≥90.0%), *n*-butyric acid (>99.5%), *n*-hexanoic acid (≥99.5%), ethyl acetate (≥99.7%), ethyl lactate (≥99.0%), ethyl butyrate (≥99.5%), ethyl hexanoate (>99.0%), and sodium heparin (Grade I-A, ≥180 USP units/mg) were all purchased from Sigma-Aldrich (St. Louis, MO, USA).

### 3.2. Animals

Adult male Kunming mice weighing 18 to 22 g were purchased from the Experimental Animal Center in Guangzhou University of Chinese Medicine. The animals were housed in top-ventilated cages under controlled conditions with a 12 h light/dark cycle and 60 ± 5% relative humidity at 25 to 28 °C. The mice were given standard rodent chow and water ad libitum for 1 week, and the animals were fasted overnight before the experiments. This study was approved by the Department of Science and Technology, Guangdong Province (Permission License No. SYXK-Yue, 2012-0125), and all of the animal experiments were performed in accordance with Guangdong Provincial Regulations for the Administration of Affairs Concerning Experimental Animals (officially issued on 1 October 2010) during the light/dark cycle between 9:00 a.m. and 6:00 p.m.

### 3.3. Preparation of the FLs

FLs with formulas similar to those of commercial Chinese liquor products were prepared by blending fusel alcohols with ethanol, organic acids, and ethyl esters. The chemical composition of the organic acids and their ethyl esters were determined by calculating their average content in 13 commercial Chinese liquor products (Table 1). The specific ratios of four major organic acids (acetic acid/l-lactic acid/*n*-butyric acid/*n*-hexanoic acid) and their corresponding ethyl esters (ethyl acetate/ethyl lactate/ethyl butyrate/ethyl hexanoate) were 3:2.5:1:1.5 and 5:6:1:6, respectively. The overall concentration of 1-propanol in the FLs was set to 0.25 g/L, according to the average value in high-quality commercial liquors. The ethanol concentration of the FLs was adjusted to 53% (*v*/*v*).

The ratio and concentration of fusel alcohols in the FLs were designated for three experiments (Table 2). In experiment A, five FLs (A1–A5) were prepared to investigate the effect of the RAAEs on the ID of the liquor product. The ratios were selected according to six commercial Chinese liquor products with different flavor types (Table 1). The total fusel alcohol content was 1 g/L, and the IA/IB ratio was 3:1. In experiment B, six FLs (B1–B6) were prepared to investigate the effect of the fusel alcohol concentration on the ID of the liquor product. Fusel alcohol concentrations of 0.5, 1.5, and 2.5 g/L were selected for two RAAEs (the optimal RAAE and the pessimal RAAE), and the IA/IB ratio remained unchanged. In experiment C, six FLs (C1–C6) were prepared to investigate the effect of different IA/IB ratios on the ID of the FLs. The optimal RAAE and the optimal fusel alcohol concentration were selected.

### 3.4. Analysis of ID

#### 3.4.1. Alcohol Feeding of Model Animals

Mice were randomly grouped for each FL sample with six mice in each group. The FLs (Table 2) were orally administered to the mice using a gavage needle at different dosages (0.356, 0.435, 0.514, 0.593, 0.672, and 0.751 g of alcohol per 100 g body weight) [21]. Alcohol feeding was conducted only once for each mouse.

#### 3.4.2. Tests of Behavioral Reactions

The animal behavioral reactions were examined prior to and after the FL feeding treatments. Tests of behavioral reactions, including rotary running and forced-swim abilities, were conducted at two different time points, that is, at 0.5 and 2 h after the feeding, which represented the rapidly increasing and slowly increasing intoxication states, respectively. The running and forced-swim tests were measured with a rotating shaft (15 cm in diameter) and an annular maze (550 mm × 350 mm × 200 mm) containing water, respectively [21]. All of the measurements were performed in triplicate.

#### 3.4.3. Assays of Blood Alcohol Concentrations (BAC)

A 100 μL blood sample was collected from the tail of the mouse and immediately transferred into a 1.5 mL tube containing 500 μL of acetonitrile, 50 μL of 500 mg/L tertiary butanol, 10 μL of sodium heparin, and 340 μL of water. The mixture was centrifuged at 12,000 r min^−1^ for 10 min (TGL-16H, Heima Med-Equipment Co., Ltd., Zhuhai, China), and then, the suspension was filtered with a 0.22 μm nylon membrane (Sterlitech, Kent, Washington, DC, USA).

The BAC was determined by the modified method of Wu et al. [21]. A Shimadzu GC system (2014 C, Shimadzu Corporation, Tokyo, Japan) equipped with a gas chromatography (GC) column (30 m × 0.25 mm × 0.25 μm, WondaCap WAX, Shimadzu Corporation, Tokyo, Japan) and a flame ionization detector (FID; Shimadzu Corporation, Tokyo, Japan) was employed for the GC analysis. The temperatures of both the inlet and the detector were maintained at 250 °C. The program of the column temperature was 48 °C for 2 min, increasing to 55 °C at a rate of 3 °C min^−1^, increasing to 200 °C at a rate of 30 °C min^−1^, and then incubating at 200 °C for 2 min. Nitrogen (high purity, 99.99%) was used as a carrier gas at a flow rate of 1.43 mL/min. The split ratio of the injector was 10:1.

#### 3.4.4. Evaluation of ID

The ID was evaluated according to the method described by Wu et al. [21]. A linear regression model between alcohol feeding dosages and comprehensive drunkenness degree calculated by integrating BAC and the behavioral reactions was applied to obtain a slope factor of *K*. The ratio of the *K* value of a given liquor product to that of purified alcohol was used to express the ID.

### 3.5. Statistical Analysis

All results were reported as the mean ± standard deviation (SD). Differences between the mean ID values for the different FLs were compared by one-way analysis of variance (ANOVA) or by the least significant difference (LSD) test; *p*-values of ≤0.05 and ≤0.01 were considered to be significant and highly significant, respectively. The statistical analysis was performed using the Statistical Product and Service Solutions (SPSS, v22.0, IBM Analytics, New York, NY, USA).

## 4. Conclusions

The importance of a proper RAAE was necessary for the FLs to have low IDs. Moreover, the effect of fusel alcohols on IDs was also extremely dependent on the comprehensive coordination of the characteristic minor components in the FLs. The IA/IB ratio had a strong positive correlation with the ID. These results will increase the awareness of fusel alcohols associated with health risks in commercial liquor products and will guide the commercial production of liquor products with a low ID.

## Figures and Tables

**Table 1:** Characteristic compounds in 13 commercial Chinese liquors.

**Compounds**	**Jiang-Flavor**		**Strong Aromatic**						**Mild Aromatic**	**Rice Aromatic**	**Te-Flavor**	**Feng-Flavor**	
	**Lang Liquor**	**Moutai**	**Jian Nan Chun**	**Wu Liang Ye**	**Wan Li Chun**	**Wan Xian Daqu**	**Shui Jing Fang**	**Yuchan Daqu**	**Fen Liquor**	**Sanhua Liquor**	**Site Liquor**	**Taibai Liquor**	**Xifeng Liquor**
Esters	297 ^a^	329	530	520	455.17	224.42	499.5	376.19	570	126	249.2	382.59	196.4
Ethyl hexanoate	23.3	42.4	216.4	198.4	154.5	66.8	-	123.5	2.2	1.71	120.1	125.5	80.3
Ethyl lactate	110.7	137.8	136.4	135.4	161.7	88.7	-	125.9	261.2	46.2	32.8	124	34.3
Ethyl acetate	105.8	147	101.7	126.4	91.33	42.9	-	81	305.9	42.1	68.4	87.9	38.3
Ethyl butyrate	21.2	26.1	40.2	20.5	13.2	6.8	-	15	-	0.6	15	13.3	15.4
Ethyl formate	-	21.2	-	-	-	-	-	-	-	-	-	-	-
Butyl acetate	-	-	-	-	-	-	-	-	-	-	-	10.4	-
Ethyl heptanoate	-	-	-	-	-	-	-	-	-	-	12.9	-	28.1
Ethyl palmitate	-	30.1	-	-	-	-	-	-	30.5	50.2	-	-	-
Acids	176	208	140	134	129.06	107.56	321.6	97.31	124	85	160	170.87	145.6
*n*-Hexanoic acid	10.2	21.8	29.1	29.6	29.8	21.9	-	17	0.2	-	-	39.8	-
l-Lactic acid	62.3	105.7	21	25.7	28.2	19.4	-	17.3	28.4	48.7	-	36.9	-
Acetic acid	76.3	11	54.6	46.5	52.9	51	-	41.8	94.5	33.9	-	67.1	-
*n*-Butyric acid	14.8	20.3	34.3	10.4	12.2	9.8	-	8.1	1.1	2.4	-	13.8	-
Alcohols	179	261	114	97	80.8	72.8	108.9	71.4	80	83	81.2	88.3	66.9
Isoamyl alcohol	45.1	49.6	34.9	34.1	32.2	34.5	38	33.3	54.6	57.8	2.1	40.2	21
Isobutanol	17.2	17	18.3	10.5	11.3	1.08 (10.8)	16.1	9.4	11.6	37.4	8.2	10.7	6.6
1-Propanol	71.1	22	23.6	17.1	20.2	20.2	-	14.9	9.5	15.7	-	16.6	-
*n*-Butanol	-	-	34.3	7	-	-	19.7	-	-	-	-	-	21.7
2-Butanol	12.8	-	6.8	5.5	-	-	-	-	3.3	-	66.5	-	13.2
1-Hexanol	-	-	12.7	6.4	-	-	-	-	-	-	-	-	-
Methanol	-	21	10.6	9.3	14	9.6	17.6	13.4	17.4	6.5	-	-	-
*β*-Phenylethanol	-	-	-	-	-	-	-	-	-	33.2	-	-	-
Aldehydes	83	111.8	70	65					14	11	10.1	-	21.9
Acetaldehyde	57.4	55	58	35.5	43.5	38.8	50.2	37.5	14	4.4	1.9	-	13.7
Acetal	15.5	7	108.8	46.8	64.5	41.5	38.3	46.4	51.4	4	8.2	-	-
Isovaleraldehyde	-	9.8	-	-	-	-	-	-	-	-	-	11	-
Furfural	-	29.4	-	-	-	-	-	-	-	-	-	-	-
	**Jiang-Flavor**		**Strong Aromatic**						**Mild Aromatic**	**Rice Aromatic**	**Te-Flavor**	**Feng-Flavor**	
RAAE ^d^	1:1:1.7		1:1.5:5.3, (1:3:4.6, Shui jing fang)						1:1.6:7.1	1:1:1.5	1:2:3.1	1:2.2:3.0	
Fusel alcohols ^b^	0.62	0.67	0.53	0.45	0.44	0.35	0.54	0.43	0.66	0.95	0.10	0.51	0.28
IA/IB ^c^	2.6	2.9	1.9	3.2	2.8	3.2	2.3	3.5	4.7	1.5	0.25	3.8	3.2

^a^ Values are expressed as mg/100 mL of commercial liquor. ^b^ The fusel alcohol units are g/L. ^c^ IA: isoamyl alcohol; IB: isobutanol; IA/IB: isoamyl alcohol/isobutanol; ^d^ RAAE: the ratio of alcohols/acids/esters; “-” represents not available.

**Table 2:** Composition, concentration, and proportion of FLs designated for three experiments.

	Concentration or Proportion					
**Experiment A**						
	A1	A2	A3	A4	A5	
RAAE	1:1:1.5	1:1.5:5	1:1.5:7	1:2:3	1:3:5	
Fusel alcohols (g/L)	1.0	1.0	1.0	1.0	1.0	
IA/IB ^a^	3	3	3	3	3	
**Experiment B**						
	B1	B2	B3	B4	B5	B6
RAAE	Optimal RAAE ^b^			Pessimal RAAE ^b^		
Fusel alcohols (g/L)	0.5	1.5	2.5	0.5	1.5	2.5
IA/IB	3	3	3	3	3	3
**Experiment C**						
	C1	C2	C3	C4	C5	C6
RAAE	Optimal RAAE					
Fusel alcohols (g/L)	Optimal level ^c^					
IA/IB	0.25	0.5	1.5	2.5	3.5	4.5

^a^ IA: isoamyl alcohol; IB: isobutanol; IA/IB: isoamyl alcohol/isobutanol. ^b^ “Optimal RAAE” and “pessimal RAAE” indicate the corresponding ratios of alcohols/acids/esters with the lowest ID and the highest ID shown in experiment A, respectively. ^c^ “Optimal level” indicates the concentration of fusel alcohols with the lowest ID shown in experiment B.

**Table 3:** IDs of FLs with different RAAEs.

FL	^i^ RAAE	^ii^ ID
A1	1:1:1.5	^iii^ 1.294 ± 0.018 ^a^ ^A^
A2	1:1.5:5	1.270 ± 0.031 ^a^ ^A^
A3	1:1.5:7	1.102 ± 0.012 ^b^ ^B^
A4	1:2:3	1.020 ± 0.018 ^c^ ^B^
A5	1:3:5	1.120 ± 0.089 ^b^ ^B^

^i^ RAAE: the ratio of alcohols/acids/esters. ^ii^ ID: intoxicating degree. ^iii^ Each ID values is expressed as mean ± standard deviation (*n* = 3). Values with different small letters (a, b, c) and with different capital letters (A, B) are significantly different (*p* < 0.05) and highly significantly different (*p* < 0.01), respectively.
